# Characterization of Resistance Mechanisms in Faba Bean (*Vicia faba*) against Broomrape Species (*Orobanche* and *Phelipanche* spp.)

**DOI:** 10.3389/fpls.2016.01747

**Published:** 2016-11-22

**Authors:** Diego Rubiales, Maria M. Rojas-Molina, Josefina C. Sillero

**Affiliations:** ^1^CSIC, Institute for Sustainable AgricultureCórdoba, Spain; ^2^IFAPA, Centro Alameda del ObispoCórdoba, Spain

**Keywords:** breeding, disease resistance, grain legume, parasitic weed

## Abstract

Faba bean (*Vicia faba*) production in Mediterranean and Near East agriculture is severely constrained by broomrape infection. The most widely distributed broomrape species affecting faba bean is *Orobanche crenata*, although *O. foetida* and *Phelipanche aegyptiaca* are of local importance. Only moderately resistant cultivars are available to farmers. Rizotrons studies allowed the dissection of resistance components in faba bean accessions against the very infective species *O. crenata, O. foetida* var. *broteri* and *P. aegyptiaca*, and to the inappropriate *P. ramosa* and *O. foetida* var. *foetida*. Results confirm that some levels of incomplete resistance are available, resulting in a reduced number of broomrape tubercles successfully formed per faba bean plant. Interestingly, the intermediate levels of resistance of cv. Baraca were operative against all broomrape populations and species studied, confirming previous reports on the stability of resistance of Baraca in field trials in different countries. Low induction of seed germination played a major role in the resistance against the inappropriate *O. foetida* var. *foetida* but not against the also inappropriate *P. ramosa*, neither to the infective species *O. crenata, O. foetida* var. *broteri*, or *P. aegyptiaca*. Negative tropism of germinated seeds with radicles growing away from faba bean roots was marked for both inappropriate species but was not observed in any of the infective species. Also, a proportion of radicles that had successfully contacted faba bean roots became necrotic, failing in starting tubercle development, particularly frequent for the two inappropriate species. Such necrosis was significant also on radicles contacting resistant faba bean accessions, being particularly relevant for Spanish *O. crenata* population, and lower although still significant in some accessions against Syrian *O. crenata* and *P. aegyptiaca*, suggesting that this might also be an operative mechanism to be selected and further exploited in faba bean resistance breeding. Even formed broomrape tubercles might later become necrotic, particularly in the case of some of the resistant faba bean accessions to the Spanish *O. crenata* and to *P. aegyptiaca* but not to the very infective Syrian *O. crenata* or *O. foetida* var. *broteri*.

## Introduction

Faba bean (*Vicia faba* L.) is a temperate grain legume of high importance as human food and animal feed (Rubiales, 2010). However, faba bean cultivation has declined in last 50 years due to low yields (Rubiales and Mikic, 2015), being broomrapes a major concern in the Mediterranean Basin and west Asia (Pérez-de-Luque et al., 2010; Maalouf et al., 2011).

Crenate broomrape (*Orobanche crenata* Forsk.) is the most damaging and widespread species infecting faba bean (Parker, 2009; Rubiales and Fernández-Aparicio, 2012; Fernández-Aparicio et al., 2016). Other species such as fetid broomrape (*O. foetida* Poir. var. *broteri*) or Egyptian broomrape [*Phelypanche aegyptiaca* (Pers.) Pomel (syn. *O. aegyptiaca* Pers.)] can be of local importance. *O. foetida* var. *broteri* is a major concern on faba bean in Beja region of Tunisia (Kharrat et al., 1992). On the contrary, *O. foetida* var. *foetida* and var. *lusitanica* are widely distributed in natural habitats in the Western Mediterranean area parasitizing wild herbaceous leguminous plants belonging to the genera *Anthyllis, Astragalus, Ebenus, Lotus, Medicago*, and *Trifolium* but not legume crops (Pujadas-Salvá, 2002; Vaz Patto et al., 2008). *P. aegyptiaca* is a very damaging species on vegetable crops prevalent in the Eastern Mediterranean and Near East that can also affect faba bean (Parker, 2009). *P. ramosa* (L.) Pomel (syn. *O. ramosa* L.) is very similar to *P. aegyptiaca* and can only very occasionally slightly infect faba bean, although at low levels, not having raised major concerns on faba bean growers.

Broomrapes are root parasitic weeds, most of their infection process taking place underground what complicates diagnosis and control (Rubiales et al., 2009; Fernández-Aparicio et al., 2011a). The most desirable control strategy is the use of resistant cultivars (Sillero et al., 2010). However, the levels of resistance available in faba bean cultivars are low and of narrow genetic basis in spite of the many efforts made by national and international programs (Pérez-de-Luque et al., 2010; Maalouf et al., 2011; Rubiales et al., 2014). Resistance against broomrape is a particularly difficult character to asses as it is highly influenced by environmental factors (Ter Borg et al., 1994; Pérez-de-Luque et al., 2004; Rubiales et al., 2006). This, together with the polygenic nature of the trait has made selection more difficult and has slowed down the breeding process (Román et al., 2002; Rubiales et al., 2009; Gutiérrez et al., 2013).

Resistance to parasitic weeds is a multicomponent trait, resulting from a battery of escape and resistance mechanisms alone or in combination (Rubiales, 2003; Pérez-de-Luque et al., 2005a,b). There is a need to find new sources of resistance, and to understand the underlying resistance mechanisms in order to facilitate faba bean resistance breeding. Dissecting the possible specific mechanisms that might be acting at different stages of the infection process might allow their combination by breeding in a single genotype resulting in a resistance more likely to be durable. The main objective of the present study was to dissect the components of the resistance of six faba bean accessions selected for their different level of resistance against different broomrape species and populations.

## Materials and Methods

### Plant Material

Six faba bean accessions (cv. Baraca, cv. Prothabon, ILB4347, ILB4350, ILB4351, and VFM26) were screened for resistance against various broomrape species under controlled conditions. Baraca is a cultivar with intermediate resistance to *O. crenata* derived from ICARDA accession Giza 402 that is so far the most widely deployed source of broomrape resistance in any faba bean breeding program (Pérez-de-Luque et al., 2010; Sillero et al., 2010). Accessions ILB4347, ILB4350, ILB4351 were developed at ICARDA and were identified as resistant against *O. crenata* under field conditions (Sillero et al., 1996; Maalouf et al., 2011). Accession VFM26 and cv. Prothabon were included as susceptible checks.

Six broomrape populations were used in this study. These were two different *O. crenata* populations collected from infected faba beans, one at Córdoba, Spain and another at Aleppo, Syria; two *O. foetida* populations, one of *O. foetida* var. *broteri*, collected from infected faba beans at Beja, Tunisia, and another of *O. foetida* var. *foetida* collected from infected *Astragalus lusitanicus* Lam. at Córdoba, Spain; a *P. aegyptiaca* population collected from infected chickpeas at Israel; and a *P. ramosa* population collected from infected tobacco at Granada, Spain.

### Glass Rhizotron Assays

Faba bean seeds were pre-germinated on wet fiber paper placed in Petri dishes and maintained in darkness at 4°C for 2 days and then at 20°C for 3 days. Seedlings were then placed in the rhizotron. Broomrape seeds were previously spread on glass fiber paper (GF/A Whatman), after being disinfected with bleach (2%) and Tween 20 (0,02%) for 5 min, and conditioned in darkness at 20°C for 8 days.

Due to the failures experienced before in using the standard rhizotron systems (Cubero et al., 1994; Pérez-de-Luque et al., 2005b) in the study of *Orobanche*/faba bean, a larger rhizotron system involving growing of host and parasite in sand between two glasses of 50 cm × 30 cm (Rubiales et al., 2006; Pérez-de-Luque et al., 2007) was used. This method allows following the root spatial distribution and the development of the broomrape at different depths. Two cork strips of 0.5 cm thickness were placed between both glasses on left and right side, and the lower side was sealed with porous material to allow the penetration of the water solution. The faba bean seeds were placed on the sand in the upper side of the plates, with the radicle on the sterilized ‘Whatmann’ glass fiber paper sheets containing the broomrape seeds on it. The glasses were suspended vertically in boxes in a controlled environment condition chamber, at 20°C and 14 h light–10 h darkness photoperiod, with a light intensity of 148 μmol/m^2^/s at leaf canopy.

After 30 days of incubation, broomrape seeds in close vicinity (<3 mm) to the faba bean roots were examined using a stereoscope microscope (30× magnification). Broomrape seeds germination percentage was determined by observing 200 seeds per plant and counting the number of them with a germ tube. Attachment percentage was determined by counting the broomrape seedlings in contact with the host root. Establishment percentage was determined by counting the number of germinated broomrape seeds that contacted the faba bean root and formed tubercles. Percentages of both necrotic broomrape radicles and nodules were also recorded. For *P. ramosa* and *O. foetida* radicles it was noticed that a number of broomrape radicles changed direction of growth when approaching the faba bean roots turning around to the opposite direction. This was recorded and referred to percentage of radicles with ‘negative tropism.’ After 45 days of incubation, the total number of broomrape tubercles per faba bean plant was recorded.

Statistical analysis (ANOVA) was performed with SPSS 10.0 for Windows. Percentages were angular transformed according to the formula $Y=\arcsin\left[\sqrt{\left(X\%\text{/100}\right)}\right]$.

## Results

Results presented in **Table 1** shows that faba bean accessions can be very susceptible to *O. crenata, O. foetida* var. *broteri*, and *P. aegyptiaca*, but confirm also that some levels of incomplete resistance are available, resulting in a reduced number of broomrape tubercles successfully formed per faba bean plant. Interestingly, the checks VFM26 and Baraca, selected for their susceptibility and resistance, respectively, to *O. crenata* in field trials at Cordoba were the most susceptible and resistant accessions not only to both populations of *O. crenata* but also to the other broomrape species studied. Clear differences in resistance were observed among the studied accessions. Interestingly, the intermediate levels of resistance of cv. Baracca were operative against all broomrape populations and species studied, confirming previous reports on the stability of resistance of Baraca in field trials in different countries (Rubiales et al., 2014). Accessions ILB4350, ILB4347, and ILB4351, previously selected as resistant in Spanish trials proved resistant to the Spanish *O. crenata* population in the rhizotron test, but although they were more resistant than the susceptible check to Syrian one, this resistance was significantly weaker to this isolate. Cultivar Prothabon was susceptible to Spanish population of *O. crenata* but resistant to the Syrian one. Interestingly some of these accessions (ILB4347, ILB4351, and Prothabon) displayed also some resistance against *O. foetida* var. *broteri*, and all of them (ILB4347, ILB4350, ILB4351, and Prothabon) displayed levels of resistance similar to Baraca against *P. aegyptiaca.* Conversely, Prothabon that was included in the study as check due to its known susceptibility to *O. crenata* in Spanish fields was significantly less susceptible to the Syrian isolate and to *O. foetida* var. *broteri* than VFM26, and interestingly very resistant to *P. aegyptiaca*.

**Table 1:** Total number of broomrapes per plant formed on roots of faba bean accessions.

	*Orobanche crenata*		*Phelipanche aegyptiaca*	*P. ramosa*	*O. foetida*	
Accession	Spain	Syria			*broteri*	*foetida*
VFM 26	59 a	86 a	42 a	5 a	143 a	0,1 a
Prothabon	53 a	37 bc	11 bc	1 b	68 bc	0 a
Baraca	5 c	17 c^∗^	5 bc	0 b	38 c	0 a
ILB4350	11 c	51 b^∗^	17 b	6 a	99 ab	0,2 a
ILB4347	12 b	48 b^∗^	4 bc	0 b	83 b	0 a
ILB4351	11 c	55 b^∗^	1 c	0 b	63 bc	0 a

*^a^Data with the same letter per column are not significantly different (Duncan test, *p* < 0,05).*

*^∗^Data with asterisk are significantly different between *O. crenata* populations from Spain and Syria (Duncan test, *p* < 0,05).*

Results presented in **Table 1** also show that faba bean is highly resistant to *O. foetida* var. *foetida* confirming the lack of infection ever observed in the faba bean in the field (Pujadas-Salvá, 2002; Rubiales et al., 2005; Vaz Patto et al., 2008). Some *P. ramosa* infection could occur, but at low levels.

Large differences were observed among broomrape species in the various steps of development, from seed germination to establishment on faba bean roots. The different broomrape species differed in the average germination on faba bean roots (**Table 2**), being the highest for *P. aegyptiaca* (range 58–81%) and *P. ramosa* (63–73%) followed by *O. crenata* (36–64%) and *O. foetida* var. *broteri* (45–56%), and very low for *O. foetida* var. *foetida* (4–19%), being in all cases in the levels previously described for these species (Fernández-Aparicio et al., 2009). Little genotypic differences were observed among the studied faba bean accessions on the level of induction of germination, although remarkable levels of reduced induction of germination have been reported in other faba bean germplasm (Fernández-Aparicio et al., 2012).

**Table 2:** Percentage of broomrape seeds germination on the vicinity of faba bean accessions.

	*O. crenata*		*P. aegyptiaca*	*P. ramosa*	*O. foetida*	
Accession	Spain	Syria			*broteri*	*foetida*
VFM 26	36 b	44 a	75 a	70 ab	56 a	19 a
Prothabon	64 a	37 a^∗^	78 a	68 ab	56 a	6 b
Baraca	57 a	42 a^∗^	58 a	68 ab	45 a	4 b
ILB4350	62 a	37 a^∗^	75 a	73 a	56 a	4 b
ILB4347	58 a	38 a^∗^	79 a	71 a	51 a	9 b
ILB4351	63 a	42 a^∗^	81 a	63 b	52 a	5 b

*^∗^Data with asterisk are significantly different between *O. crenata* populations from Spain and Syria (Duncan test, *p* < 0,05).*

Once germinated, broomrape radicles contacted faba bean roots in their vicinity with a level of success that varied with the species (**Table 3**), being lower in the case of *P. ramosa* (range 4–11%) but similar for *P. aegyptiaca* (21–46%), *O. foetida* var. *broteri* (30–45%), *O. foetida* var. *foetida* (15–34%). Interestingly, there were significant differences between the Syrian and the Spanish *O. crenata* population in success of seed germination (**Table 2**) and of germinated seed contact (**Table 3**), being germination in general lower for Syrian population (37–44% vs. 36–64%) but the success contacting faba bean of the germinated seeds higher (38–56% vs. 23–38%).

**Table 3:** Percentage of germinated broomrape seeds contacting roots of faba bean accessions.

	*O. crenata*		*P. aegyptiaca*	*P. ramosa*	*O. foetida*	
Accession	Spain	Syria			*broteri*	*foetida*
VFM 26	33 a	47 ab	29 bc	11 a	45 a	34 a
Prothabon	38 a	54 a	46 a	9 ab	39 ab	21 ab
Baraca	23 a	48 ab	36 ab	4 b	40 ab	23 ab
ILB4350	29 a	56 a	31 bc	10 a	45 a	28 ab
ILB4347	36 a	38 b	27 bc	7 ab	30 b	15 b
ILB4351	27 a	51 a	21 c	8 ab	32 b	23 ab

It was remarkable to observe that one of the possible mechanisms for not contacting with faba bean roots could be negative tropism, with broomrape radicles changing growing direction when closer to the faba bean roots (**Table 4**). This negative tropism could be high in the case of *O. foetida* var. *foetida* (32–70%) or *P. ramosa* (range 21–30%) but was not observed in radicles on any of the other species.

**Table 4:** Percentage of broomrape radicles showing negative tropism when approaching roots of faba bean accessions.

	*O. crenata*		*P. aegyptiaca*	*P. ramosa*	*O. foetida*	
Accession	Spain	Syria			*broteri*	*foetida*
VFM 26	0	0	0	27 a	0	55 ab
Prothabon	0	0	0	21 a	0	32 b
Baraca	0	0	0	30 a	0	40 b
ILB4350	0	0	0	22 a	0	36 b
ILB4347	0	0	0	30 a	0	70 a
ILB4351	0	0	0	26 a	0	45 ab

Broomrape radicles successfully contacting faba bean roots might later become necrotic (**Table 5**) or succeed further in establishment and develop broomrape tubercles on the roots (**Table 6**), a few of which might develop and grow further or fail and become necrotic (**Table 7**). **Table 5** shows how a proportion of the broomrape radicles became necrotic, with clear differences among species being highest for the inappropriate species that hardly infect faba bean such as *P. ramosa* (range 54–67%) and *O. foetida* var. *foetida* (31–93%). On the contrary necrosis of radicles in vicinity of faba bean roots was low for the remaining species but could be higher for some species in the vicinity of roots of the resistant accessions. This was particularly evident for the Spanish *O. crenata* population with 20–28% of necrotic radicles on resistant accessions vs. 1% on the susceptible checks.

**Table 5:** Percentage of broomrape radicles contacting faba bean roots that became necrotic.

	*O. crenata*		*P. aegyptiaca*	*P. ramosa*	*O. foetida*	
Accession	Spain	Syria			*broteri*	*foetida*
VFM 26	1 b	0 b	0 c	57 a	0 b	61 b
Prothabon	1 b	3 b	2 c	54 a	10 a	70 ab
Baraca	24 a	12 a	14 a	58 a	1 b	31 b
ILB4350	28 a	5 b	3 bc	66 a	1 b	64 ab
ILB4347	20 a	5 b	8 ab	67 a	0 b	88 a
ILB4351	24 a	13 a	3 bc	63 a	4 ab	93 a

**Table 6:** Percentage of germinated broomrape seeds that successfully established a tubercle on faba bean roots.

	*O. crenata*		*P. aegyptiaca*	*P. ramosa*	*O. foetida*	
Accession	Spain	Syria			*broteri*	*foetida*
VFM 26	80 a	75 bc	46 a	34 a	86 ab	1 a
Prothabon	57 ab	86 ab	55	6 bc	76 ab	0 a
Baraca	22 c	68 c	20 b	0 c	84 ab	0 a
ILB4350	33 bc	82 ab	40 a	26 ab	93 ab	4 a
ILB4347	48 abc	87 a	7 b	0 c	97 a	0 a
ILB4351	41 bc	77 abc	0 c	0 c	74 b	0 a

**Table 7:** Percentage of established broomrape tubercles that became necrotic.

	*O. crenata*		*P. aegyptiaca*	*P. ramosa*	*O. foetida*	
Accession	Spain	Syria			*broteri*	*foetida*
VFM 26	2 c	4 a	0 b	6 a	2 a	–
Prothabon	0 c	0 a	1 b	0 a	1 a	–
Baraca	23 b	8 a	23 a	0 a	1 a	–
ILB4350	55 a	11 a	0 b	0 a	0 a	–
ILB4347	38 ab	1 a	0 b	0 a	4 a	–
ILB4351	2 c	8 a	0 b	0 a	2 a	–

Success in tubercle development is species dependent (**Table 6**), being particularly high for the Syrian *O. crenata* population (range 68–87%) and for *O. foetida* var. *broteri* (76–97%) but negligible for *O. foetida* var. *foetida*. Clear genotypic effects were observed by some faba bean accessions for the Spanish *O. crenata* population (range 22–80%) or even for the inappropriate *P. ramosa* (0–34%).

A proportion of these formed broomrape tubercles might later become necrotic, particularly in the case of some of the resistant faba bean accessions to the Spanish *O. crenata* (accessions Baraca, ILB4350 and ILB4347) and to *P. aegyptiaca* (Baraca) but not to the Syrian *O. crenata, P. ramosa*, or *O. foetida* var. *broteri*.

## Discussion

The resistance to broomrape in faba bean is scarce and of complex nature what complicates resistance breeding (Rubiales et al., 2006; Sillero et al., 2010). Most resistant cultivars have been bred using the Egyptian line G402 as the major donor of resistance that derives mainly from resistant breeding lines developed at ICARDA, Syria, selected across different Mediterranean countries, therefore, it is not surprising that the resistance is operative against different *O. crenata* populations. We show here that this resistance is broadly operative not only against diverse *O. crenata* populations but also against other species such as *O. foetida* and *P. aegyptiaca*. Still, relying on a single source of resistance is risky and broadening the genetic basic of resistances deployed is a major need in order to increase durability of resistance.

Little conclusive information has been reported on the nature of the resistance available in faba bean. Existing reports points toward resistance hampering establishment and development of broomrape tubercles (Khalaf and El-Bastawesy, 1989; Pérez-de-Luque et al., 2007, 2010) what confirms some of our findings (**Tables 6 and 7**). We found in this study little variation for the induction of broomrape seed germination among the studied accessions (**Table 2**), what is in agreement with most previous reports that considered this non-existent in faba bean against both *O. crenata* and *O. foetida* (Ter Borg et al., 1994; Pérez-de-Luque et al., 2010). However, resistance based in no-induction of broomrape seed germination has been recently described and characterized in other germplam (Fernández-Aparicio et al., 2012) and will be most useful in faba bean resistance breeding.

Clear differences in germination were observed between the two *O. foetida* populations. This reduced germination might be related to a low secretion of specific germination stimulants in the faba bean roots. A host specialization process, which is still in progress, has been suggested for *O. foetida* (Román et al., 2007; Vaz Patto et al., 2008). Its specificity has been related to their sensitivity to non-strigolactone compounds such as peagol and a polyphenol (Evidente et al., 2009, 2010). Contrary to the other species, neither *O. foetida* var. *broteri* nor var. *foetida* respond to the synthetic strigolactone GR24 (Fernández-Aparicio et al., 2009), widely used as standard germination stimulant, but it does respond to other strigolactones such as fabacyl acetate known to be produced in faba bean (Fernández-Aparicio et al., 2011b). Host specialization might be mediated by the combination in the root exudate of a number of signaling chemicals affecting germination. Separately these chemicals might have a stimulatory (various strigolactones but also other metabolites) or inhibitory effect (Whitney, 1978; Evidente et al., 2007; Fernández-Aparicio et al., 2008), but combined they might have synergistic or antagonistic effects.

Low induction of seed germination seemed to play a major role in the non-host resistance against the inappropriate (little infective) species *O. foetida* var. *foetida* (<20% germination) but not against the also inappropriate *P. ramosa* (>63%, similar to that of infective species). Although not identified in any of the faba bean accessions included in this study, low induction of germination can also play a significant role in host resistance and has been reported in other accessions (Fernández-Aparicio et al., 2012, 2014), in chickpea against *O. crenata* (Rubiales et al., 2003), and in sorghum against *Striga* (Ejeta, 2007).

A second mechanism was observed only in radicles of the inappropriate species, showing ‘negative tropism’ with radicles turning growing direction away of faba bean roots, being as high as 32–70% for *O. foetida* var. *foetida* and for 21–30% for *P. ramosa*. This was not observed in any of the infective species. This might be ascribed to exudation of metabolites by faba bean roots having a negative effect on growth of radicle of these species, but we cannot exclude overproduction of stimulants as directional growth of broomrape radicles is a response to germination stimulants gradient (Whitney and Carsten, 1981). We did not measure radicle length, but noticed a reduced success rate of radicles of *P. ramosa* to contact faba bean roots, what in addition to the above described negative tropism might be ascribed to limited growth, what points toward inhibition. This deserves further investigations.

Also, a proportion of radicles that had successfully contacted faba bean roots became necrotic, failing in starting tubercle development, what also suggest a response to chemicals exuded by faba bean roots. This necrosis of radicles was particularly frequent for the inappropriate *P. ramosa* and *O. foetida* var. *foetida* suggesting a major role of inhibitory/toxic chemicals on this non-host resistance. Such necrosis was significant also on radicles contacting resistant faba bean accessions, being particularly relevant for Spanish *O. crenata* population, and lower although still significant in some accessions against Syrian *O. crenata* and *P. aegyptiaca*, suggesting that this might also be an operative mechanism to be selected and further exploited in faba bean resistance breeding.

Even formed broomrape tubercles might become necrotic, particularly in the case of some of the resistant faba bean accessions to the Spanish *O. crenata* (accessions Baraca, ILB4350 and ILB4347) and to *P. aegyptiaca* (Baraca) but not to the very infective Syrian *O. crenata* or *O. foetida* var. *broteri*. Zaitoun et al. (1991) and Zaitoun and Ter Borg (1994) already suggested a barrier in the roots of Baraca leading to the death of *O. crenata* in its early developmental stages. This response has frequently been reported also in other plants against various parasitic weeds, histology revealing that initial vascular connections are established but are then blocked by accumulation of mucilage, secretions and degraded products not allowing nutrient flux between host and parasite, causing death of tubercles at an earlier stage (Labrousse et al., 2001; Zehhar et al., 2003; Pérez-de-Luque et al., 2005b, 2006).

The existence of races of *O. crenata* has remained a controversial issue, with little conclusive reports supporting this. Although variation among *O. crenata* populations in the ability to parasitize different faba bean accessions have been suggested (Radwan et al., 1988), no physiological races have been reported. It is concluded from our study that the Syrian *O. crenata* population is more aggressive than the Spanish one, causing more infection than the Spanish population on all resistant accessions, but in all cases, infection was significantly lower than on the check VFM26. However, the differential response of cv. Prothabon, being susceptible to the Spanish population but resistant to the Syrian one, might point toward some variation for virulence among populations. However, these results are too preliminary as to claim for existence of races in *O. crenata*, what should be further studied. However, it might well be possible that more virulent biotypes could be selected when challenged by the widespread use of newly deployed highly resistant cultivars. This deserves constant monitoring.

The fact that the resistance of Baraca to the Spanish *O. crenata* population is also effective against the Syrian one is not surprising, as Baraca derives from resistant breeding lines developed at ICARDA, Syria, having line G402 as the major donor of resistance in its pedigree. This breeding material has been the foundation of most faba bean breeding programs for broomrape resistance and it seems that is in the pedigree of most, if not all, registered faba bean resistant cultivars. This resistance, having been selected in various countries, seems to be broadly effective. Only long term cultivation of these cultivars in large areas will say if it is indeed durable or will be easily overruled by the appearance of a most virulent *O. crenata* population. Time is needed to verify this, as these cultivars are relatively recent and are not very widely deployed. For instance, cultivation of cv. Baraca is still negligible in Spain. All we can speculate is that, the remarkable fact that this resistance is not only operative against different populations of *O. crenata*, but also against different species such as *O. foetida* (confirming field studies of Rubiales et al., 2014) and *P. aegyptiaca*, suggesting that Baraca carries broad sense resistance, likely to be durable.

## Conclusion

Results presented here show the broad base of the resistance of cv. Baraca, being effective not only against contrasting *O. crenata* populations but also against other species such as *O. foetida* and *P. aegyptiaca*. Dissecting specific resistance mechanisms acting at different stages of the infection process will facilitate their combination in a single genotype by breeding and selection providing a resistance more likely to be durable.

## Author Contributions

MR-M performed the experiments supervised by JS and DR who wrote the manuscript.

## Conflict of Interest Statement

The authors declare that the research was conducted in the absence of any commercial or financial relationships that could be construed as a potential conflict of interest.
